# Cutting blade dentitions in squaliform sharks form by modification of inherited alternate tooth ordering patterns

**DOI:** 10.1098/rsos.160385

**Published:** 2016-11-30

**Authors:** Charlie Underwood, Zerina Johanson, Moya Meredith Smith

**Affiliations:** 1 Department of Earth and Planetary Sciences, Birkbeck, University of London, London, UK; 2 Department of Earth Sciences, Natural History Museum, London, UK; 3 Dental Institute, Craniofacial Development, King's College London, London, UK

**Keywords:** sharks, *Squalus*, squaliforms, evolution of teeth, replacement pattern

## Abstract

The squaliform sharks represent one of the most speciose shark clades. Many adult squaliforms have blade-like teeth, either on both jaws or restricted to the lower jaw, forming a continuous, serrated blade along the jaw margin. These teeth are replaced as a single unit and successor teeth lack the alternate arrangement present in other elasmobranchs. Micro-CT scans of embryos of squaliforms and a related outgroup (Pristiophoridae) revealed that the squaliform dentition pattern represents a highly modified version of tooth replacement seen in other clades. Teeth of *Squalus* embryos are arranged in an alternate pattern, with successive tooth rows containing additional teeth added proximally. Asynchronous timing of tooth production along the jaw and tooth loss prior to birth cause teeth to align in oblique sets containing teeth from subsequent rows; these become parallel to the jaw margin during ontogeny, so that adult *Squalus* has functional tooth rows comprising obliquely stacked teeth of consecutive developmental rows. In more strongly heterodont squaliforms, initial embryonic lower teeth develop into the oblique functional sets seen in adult *Squalus*, with no requirement to form, and subsequently lose, teeth arranged in an initial alternate pattern.

## 1. Introduction

**Figure 1.**
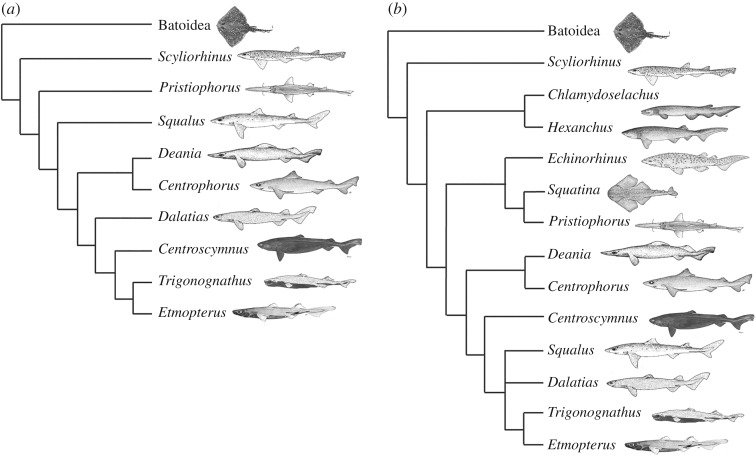
Competing recent molecular phylogenies showing relative position of included taxa. All taxa mentioned in the text are included to give an overview of the position of both squaliforms and non-squaliform squalean sharks. Note the discrepancy in the relative positions of *Squalus* in Straube *et al.* [5] and Naylor *et al.* [3]. Images not to scale; slightly modified from FAO publications under a Creative Commons Attribution-Noncommercial 3.0 license; *Trigonognathus* redrawn from various sources.

**Figure 2.**
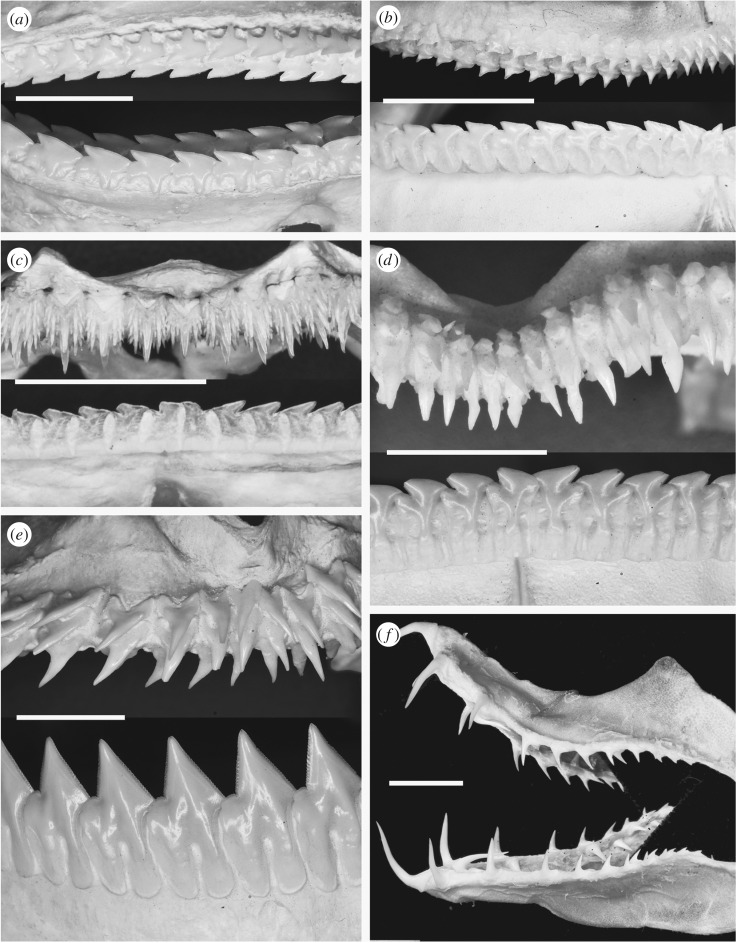
Dentitions of squaliform sharks. All images of labial views of the dentitions. Scale bars all 10 mm. (*a*) Upper and lower dentition of *Squalus acanthias*; mature specimen, North Sea. (*b*) Upper and lower dentition of *Centrophorus* sp.; species uncertain, Philippines. (*c*) Upper and lower dentition of *Etmopterus pusillus*; mature specimen, Azores. (*d*) Upper and lower dentition of *Centroscymnus coelolepis*; mature specimen, Azores. (*e*) Upper and lower dentition of *Dalatias licha*; mature specimen, Azores. (*f*) Upper and lower dentition of *Trigonognathus kabeyai*; mature specimen, Japan.

The squaliform sharks form a monophyletic clade containing a quarter of all shark species. They have a cosmopolitan body form but vary greatly in size from some of the smallest of all sharks, to species of *Somniosus* reaching over 6 m in length. They are widely distributed in marine environments, but are especially diverse in deep marine habitats, where they are commonly the dominant chondrichthyan group. The squaliform dentition is extremely variable (e.g. [1,2]), with virtually all tooth morphotypes present within squaliforms limited to the clade. Although some plesiomorphic characters, such as fin spines, are present, the squaliforms are derived relative to many other shark clades (e.g. [3–5]; figure 1), and appear later in the fossil record than most other extant clades [1,6]. Despite the derived phylogenetic position of the squaliforms, and the variety of dentitions that they possess (figure 2), the widespread squaliform *Squalus acanthias* is commonly used as a ‘model organism’ for Chondrichthyes (e.g. [7]) due to its wide distribution and abundance. Although the skeletal anatomy of this species is extremely well documented (e.g. anatomical dissection guides), the dentition has received scant interest. Adult *Squalus* possess teeth forming a single row along the jaw margin, with adjacent teeth overlapping via a flange on the lateral part of the root, resulting in the formation of a continuous serrated blade along the occlusal jaw edge.

**Figure 3.**
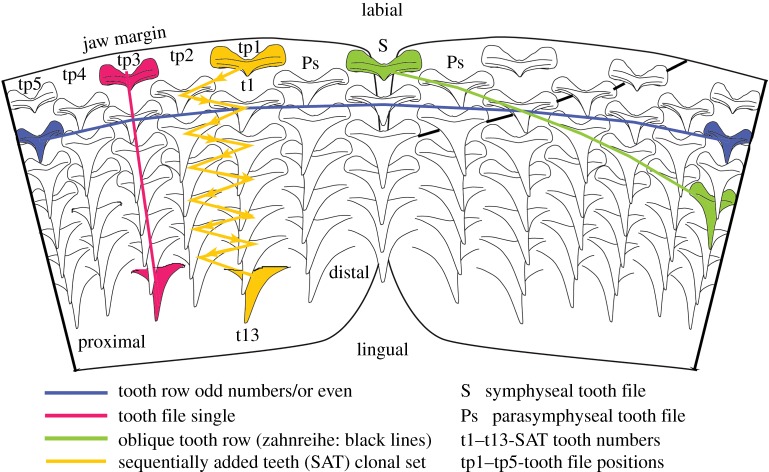
Diagrammatic view of the developing teeth within an elasmobranch jaw with alternate file tooth replacement, showing the terminology used here. t1–13, numbers of teeth in one SAT unit; tp1–5, disto-proximal tooth positions; Ps, parasymphysial; S, symphysial.

The dentition for each clade of gnathostomes, including Chondrichthyes, is unique, and we examine how a common developmental model [8] is expressed within neoselachians (the clade including all living sharks and rays), and particularly squaliforms. The formation of a deep, continuous sub-epithelial dental lamina, distinctive for neoselachians but not osteichthyans [9], promotes regulated tooth development along the lingual jaw margin; tooth addition patterns distinctive for sharks [10] and for rays [11] are overprinted onto this. Teeth are produced only within the dental lamina, and tooth germs migrate in a time- and space-controlled mechanism towards their functional position at the jaw margin. Here, timing of tooth shedding, or retention may be variable ([8,12]; figure 3). These concepts predict that certain shark dentition morphologies can be interpreted as derived from a sequential addition model (SAM) [8,10], where development of integrated tooth addition was based on a clonal set (teeth generated as two adjacent files with alternate timing and arrangement as a sequential addition tooth (SAT) module; figure 3). This was envisaged as a developmental segment of the dentition iteratively repeated in a proximal direction along the jaw, so that alternate replacement of teeth was controlled by the initiation pattern for each pair of tooth files [8]. This produced the alternate dentition, where teeth in subsequent rows (parallel to the jaw margin; figure 3) were offset by one jaw position from each other. A different scheme of non-alternate tooth replacement was described (single file), where each generative set is equivalent to one tooth file, set up by the initial tooth, in line with the central cusp of the later developing teeth in the file [8,10]. As in the alternate model, tooth size increases with developmental time within each generative set until maturity.

It was noted (e.g. [10]) that single file tooth addition characterized fewer clades than alternate, but it is unclear how these are related to each other developmentally and which pattern represents the plesiomorphic state within elasmobranchs. Alternate file tooth addition is present within all batoids [11] and probably all galean sharks (Galea, e.g. [10]). Among the squalean sharks (Squalea), alternate file tooth addition is evident in the Pristiophoridae. Single file tooth addition was said to characterize the squaliforms (including *Squalus*), as well as in *Echinorhinus* (Echinorhinidae) and, at least in the lower dentition, in the Hexanchidae. In taxa where teeth are widely spaced and/or the dentitions spread over a strongly curved jaw surface (lamniforms, *Squatina* and *Chlamydoselachus*), the addition pattern is less clear.

The squaliforms, and a number of other squalean groups, thus deviate from the typical, and probably plesiomorphic, model of alternate tooth replacement. While tooth development associated with the alternate dentition is well known [10,11], the details of single file tooth replacement have not been studied. There is, therefore, no information as to whether the single file tooth replacement pattern seen in (predominantly) squaliforms represents a fundamentally different process to that in other elasmobranchs, or a modification of the typical tooth development process leading to a radically different pattern.

Several squaliform taxa were examined to determine whether their dentitions could be described as developing from alternate or single files. Our observations were mapped onto existing cladograms (figure 1), to determine the phylogenetic distribution of these patterns, and to test whether the alternate tooth succession pattern is derived from the single, or vice versa. We also address how dental pattern can be developmentally modified during growth from the initial embryonic pattern, to generate diverse examples of dental morphologies in squaliforms from an inherited common plan.

## 2. Material and methods

Dentitions of a number of late-stage squaliform embryos were examined, including representatives of all major squaliform clades, in addition to dentitions of juveniles and adults of many taxa. Embryos were used to observe the first-formed teeth, which may be lost by the time of birth. Adults and juveniles were also studied to document ontogenetic changes in the dentitions. Specimens of embryos of the genera *Centroscymnus*, *Deania*, *Etmopterus* and *Squalus* were studied by X-ray computed tomography (XCT, table 1).

**Table 1.** Table of specimens studied by CT scanning.

species	size	scans	notes
*Centroscymnus coelolepis*	26 cm embryo	1	full squamation and small yolk sac
*Deania calcea*	21 cm embryo	1	incipient squamation and small yolk sac
*Etmopterus spinax*	10 cm embryo	1	no squamation and moderate size yolk sac
*Etmopterus spinax*	16 cm neonate/juvenile	1	smallest free swimming specimen observed
*Etmopterus spinax*	36 cm adult	1	female
*Squalus* sp.	approx. 12 cm embryo	2	no squamation and small yolk sac
			dissected without being measured
*Squalus blainville*	15 cm embryo	3	partial squamation and large yolk sac
*Squalus acanthias*	11 cm embryo	1	partial squamation and moderate size yolk sac
*Squalus acanthias*	14 cm embryo	2	no squamation and large yolk sac
*Squalus acanthias*	adult	1	jaw, no additional data
*Dalatias licha*	adult	1	jaw, no additional data

Dried and prepared dentitions of non-embryos (juveniles and/or adults) of approximately 20 additional squaliform species available to us were also studied, while an additional embryo of *Deania calcea* was studied by dissection.

Embryos of the pristiophorids *Pliotrema* and *Pristiophorus* were examined (see [13] for details of the specimens), representing a sister group to the Squaliformes within the Squalea (figure 1*b*) [3,5], with dentitions clearly showing an alternate pattern in the adult. The Pristiophoridae therefore possess the same dental patterning present in all galean sharks and batoids yet studied, and therefore preserve the probable plesiomorphic dental condition to which the Squaliformes can be compared. As noted above, dental patterning of the squaleans *Squatina* and *Chlamydoselachus* is unclear due to the wide spacing of tooth files, while the single file dentition in the lower dentition of the Hexanchidae contrasts to the alternate pattern in upper jaw and teeth that lie closest to the jaw hinges. Dental development of embryos of these additional squalean clades is currently in progress and is not part of this study.

The functional teeth of many squaliforms are arranged in a single row along the jaw margin (see below). Teeth rotate into functional position before descending the labial face of the jaw prior to being shed. As a result, any specimen lacking teeth in the functional position, or ‘post functional’ position on the labial face of the jaw cartilage, represents a developmental stage prior to the first-formed teeth being shed.

### 2.1. CT-scanning

**Figure 4.**
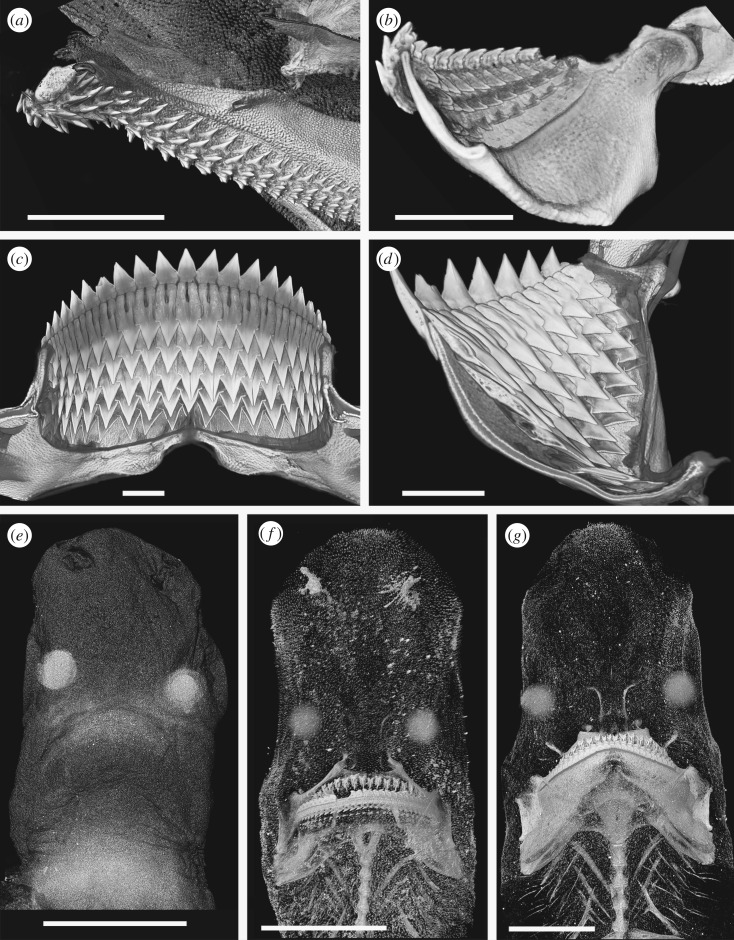
Squaliform and other shark tooth development. All images rendered micro CT data. Scale bars all 10 mm. (*a*) Virtual cross section through the upper dentition of *Scyliorhinus canicula* showing the multiple functional tooth rows*.* (*b*) Virtual cross section through the lower dentition of *Squalus acanthias.* (*c*) Virtual cross section through the lower dentition of *Dalatias licha.* (*d*) Lingual view of the replacement teeth in the lower jaw of *Dalatias licha*. (*e*) Head of an embryo of *Etmopterus spinax*; the only mineralized tissues are the tips of the teeth, but the eye lenses are also conspicuous. Northeast Atlantic. (*f*) Head of a juvenile of *Etmopterus spinax*; note the incomplete mineralization of the jaws and brachial system and lack of mineralized chondrocranium. Northeast Atlantic. (*g*) Head of an adult of *Etmopterus spinax*; note the incomplete mineralization of the chondrocranium in contrast to the strongly mineralized jaws. Northeast Atlantic.

**Figure 5.**
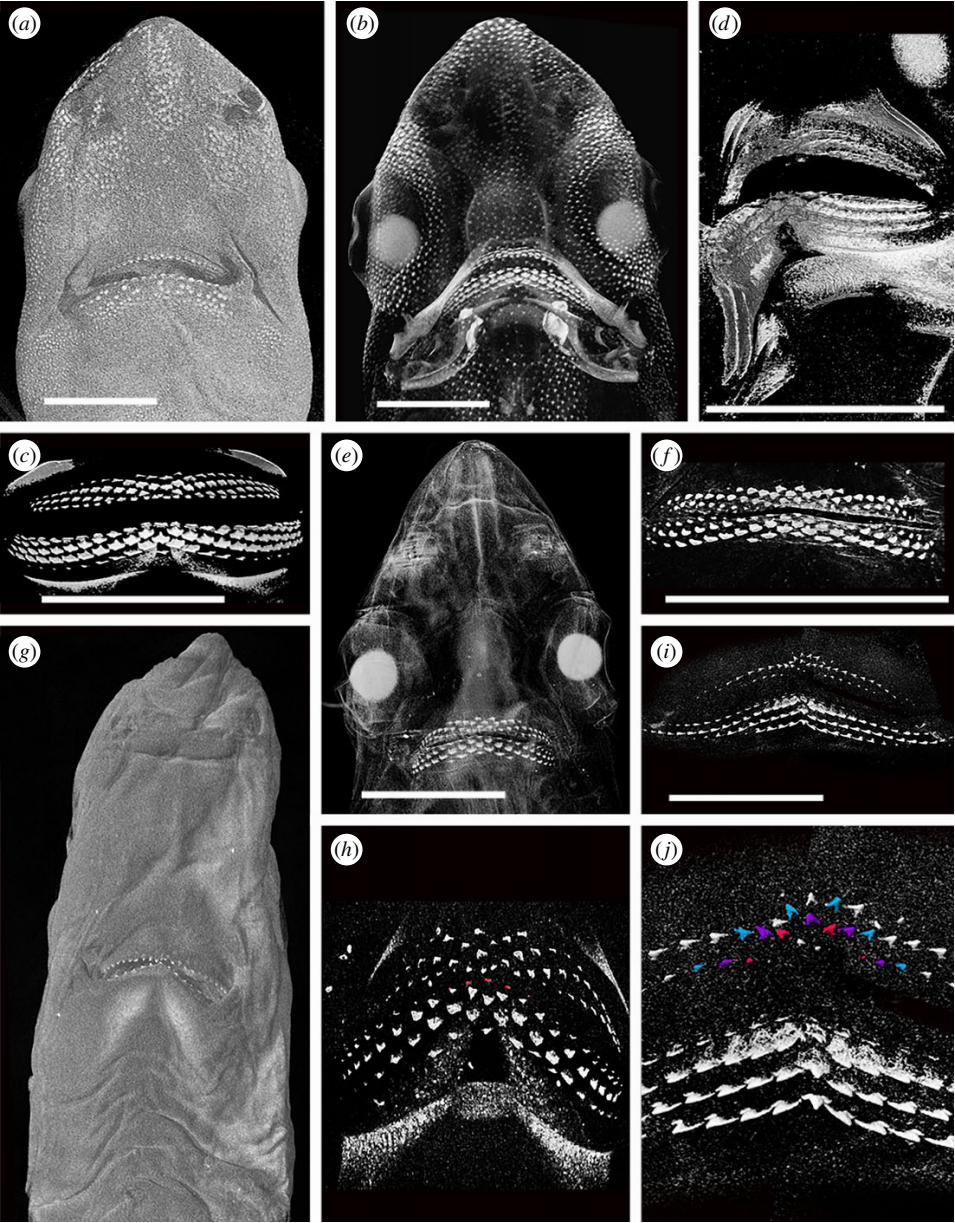
Tooth formation in *Squalus* and *Deania*. All images rendered micro-CT data. Scale bars all 10 mm. (*a*–*c*) Renders of a 15 cm embryo of *Squalus blainville*. (*a*) General view of ventral side of head. (*b*) General view of ventral side of head with greater penetration. (*c*) Lingual view of the dentition. (*d*) Render of an oblique view of the dentition of the 11 cm embryo of *Squalus acanthias*. (*e*,*f*) Renders of a 14 cm embryo of *Squalus acanthias*. (*e*) General view of ventral side of head showing the teeth as the only mineralized structure. (*f*) Lingual view of dentition. Symphyseal and parasymphyseal first lower teeth highlighted. (*g*,*h*) Renders of an approximately 12 cm embryo of *Squalus* sp. (*g*) General view of ventral side of head. (*h*) Lingual view of dentition. (*i*,*j*) Renders of a 21 cm embryo of *Deania calcea*. (*i*) Composite image of lingual view of dentition. (*j*) Detail of the symphyseal region of the dentition with alternate files highlighted in the upper dentition.

Specimens were scanned using the Metris X-Tek HMX ST 225 XCT scanner at the Imaging and Analysis Centre, Natural History Museum, London. Three-dimensional volume rendering and analyses were performed using Avizo Standard software (v. 8.0.1) (https://www.fei.com/software/amira-avizo/), VGStudio MAX v. 2.0 (http://www.volumegraphics.com/en/products/vgstudio-max.html) and Drishti (http://sf.anu.edu.au/Vizlab/drishti). The weakly mineralized teeth of some chondrichthyan embryos frequently show very little X-ray contrast to the surrounding soft tissue, sometimes showing less X-ray opacity than proteinaceous eye lenses (figures 4*e* and 5*j*), making these difficult to render. In some specimens, multiple scans and renders were made using different software and settings, and some renders are composite images using a range of settings. Segmentation was not used in cases of low degrees of mineralization due to the risk of artefacts being produced because of difficulties in isolating dental material. It should be noted that renders optimized to show the most weakly mineralized structures are often unsuitable for other purposes due to noise, and different renders of the same specimen may give the impression of different numbers of developing teeth.

## 3. Tooth arrangement in sharks: terminology

The dentition of most adult squaliforms contains blade-like teeth, either on both jaws or only on the lower jaw. These teeth typically form a single row along the jaw margin, with adjacent teeth partly overlapping at the tooth base. This creates a continuous, serrated, cutting edge along the jaw margin with lingual replacement sets aligned as single files (figure 2*a*–*e*). The dentition rotates into functional position as a single unit, and tooth rows are shed as connected suites of multiple teeth. Other teeth, typically in the upper jaw but more rarely on the lower as well, are slender and show an alternate pattern typical of other sharks and rays (figure 2*c*–*f*). The dentition of the earliest known squaliform, *Protosqualus*, comprises blade-like teeth with overlapping attachment bases on both jaws, but the bladed morphology is not as well developed as in *Squalus* and many other modern taxa [1,6].

Although the original terminology associated with arrangement of developing teeth within the dental lamina has previously been explained [8,10], use of these terms, and their applicability is clarified below, in particular for adult morphology (figure 3).

— *File*: labial to lingual set of successional teeth lingual to functional teeth at each position along the jaw (vertical row [8]; figures 3, 8 and 9). May be either arranged as single files (replacement teeth for each tooth along jaw margin, previously ‘tooth family’) or, alternate files (see SAT unit) with teeth staggered as different generation times in a clonal set at even and odd jaw positions (figure 3, yellow tp1 + tp2, t13, most lingual tooth).
— *Symphyseal file*: file on or spanning the jaw symphysis. The tooth may be polarized and asymmetrical (left or right [10]) or symmetrical (figure 3, S).
— *Parasymphyseal file*: two files of teeth on either side of the jaw symphysis, with mirror images of left and right morphologies. In alternate dentitions, will only occur in rows lacking a symphyseal tooth file (figure 3, Ps).
— *Sequentially added teeth (SAT)*: two adjacent, even and odd files repeated proximally along the jaw with an alternate file pattern of tooth succession; representing clonal developmental tooth sets [10] (SAT unit, figure 3, tp1 + tp2, yellow arrows, t1–t13).
— *Row:* includes all teeth along the adult jaw [12] comprising a single row parallel to the jaw margin (old term, horizontal row, starting from the symphysis).
— *Developmental row*: teeth along the length of the jaw that belong to the same generation (figure 3, blue).
— *Oblique row*: teeth aligned obliquely across developmental rows, each of a different developmental time (figure 3, green).
— *Functional row*: teeth along the length of the adult jaw, each in the same position relative to the jaw margin or occlusal edge. This can differ from the developmental row, by containing teeth representing different generations on the jaw margin due to asynchronous timing of development.
— *Alternate file tooth replacement*: teeth in paired adjacent files are offset (SAT) along the tooth rows. Only alternate rows possess a symphyseal tooth (where present).
— *Single file tooth replacement*: teeth in adjacent files are parallel to each other and not offset along the tooth rows. All tooth rows possess a symphyseal tooth.
— *Proximal, distal*: direction along the jaw, proximal being closer to the jaw joint, distal closer to the jaw symphysis. While these terms are standard usage in developmental studies, they have rarely been used in the context of chondrichthyan dentitions. While the terms ‘anterior’ and ‘posterior’ have commonly been used in describing chondrichthyan jaw positions, they are unsuitable due to the variation of jaw orientation in sharks and batoids. In most batoids and squaliform sharks, jaw cartilages are orientated normal to the long axis of the neurocranium during life, so that the term ‘anterior’ more accurately describes the upper dentition than the symphyseal region of either jaw.
— *Mesial, distal*: these terms refer to the parts of individual teeth, with the mesial side pointing towards the symphysis and the distal towards the commissure. While the term ‘distal’ is also used in relation to jaw position, these terms can clearly be separated by their context.

## 4. Squaliform phylogeny

The squaliform sharks form a monophyletic clade within the Squalea, one of the two main branches of living sharks. The monophyly of the Squalea is strongly supported by molecular studies (e.g. [14]). The interrelationships within the squaliforms are less clear. A wide-ranging study by Vélez-Zuazo & Agnarsson [15] placed the Squalidae as the most derived family within the clade and the Etmopteridae as the sister group to the rest of the clade. Naylor *et al*. [3] questioned the data used in this study, and presented instead a phylogeny where the Centrophoridae were the most basal family within the squaliforms, with the Squalidae forming a sister group to the Dalatiidae within the more derived forms. In contrast to these results, Straube *et al.* [4,5] used a different suite of molecular markers to Naylor *et al.* [3], concluding that *Squalus* was a sister taxon to other squaliforms, with the Centrophoridae and Dalatiidae being progressively more derived sister groups to the Etmopteridae + Somniosidae [5]. A study using both fossil and extant squaliforms by Klug & Kriwet [6] also concluded that the Squalidae were the sister group to all other squaliforms, with the Centrophoridae as the next most basal and a sister group to the more derived families. The basal position of the Squalidae is consistent with this family having the earliest appearance in the fossil record [6] and what was considered to be the least derived dental morphology, in a study of dental characters [1].

## 5. Squaliform dentitions

The teeth of squaliforms are varied, both in their morphology and the degree to which the dentition differs between upper and lower jaw (dignathic heterodonty; see below). The dentitions of some genera, such as *Squalus*, show very little dignathic heterodonty, with upper and lower teeth of similar morphology. By contrast, the majority of squaliform species possess extremely pronounced heterodonty, typically comprising larger, blade-like teeth on the lower jaw and small, pointed teeth on the upper.

The dentition of *Squalus* (figure 2*a*) is in many respects the simplest of all of the squaliform sharks, the closest among living squaliforms to dentitions of *Protosqualus*, the earliest known (fossil) member of the clade [1]. *Squalus* possesses a very low degree of dignathic heterodonty, with little variation in tooth shape within a jaw, between sexes or between different ontogenetic stages (e.g. [2]). The teeth are low and linguo-labially compressed. The crown comprises a steeply inclined and blade-like cusp over a distal heel. The distal edge of one tooth overlaps with, and fits into, an indentation within the mesial edge of the neighbouring tooth. The teeth thus form a continuous, tooth-interlocked, blade on the jaw margin. During tooth replacement, the previous row may be retained, producing a double row of functional teeth.

Within the Centrophoridae, *Centrophorus* and *Deania* have a higher degree of heterodonty than in *Squalus* (figure 2*b*). Lower teeth are broadly similar to those of *Squalus*, although the root is higher and more compressed and serrations are present on the teeth of some species. Upper teeth of *Centrophorus* and *Deania* are narrower and more erect than lowers, with a single, triangular cusp. The degree to which the cusps of the upper teeth are inclined and hence resemble the lower teeth differs between species.

Dentitions of other squaliform families typically show extreme heterodonty, although there are exceptions. In many taxa, the lower teeth have a crown that is similar to *Squalus*, but with a greatly enlarged and more compressed root (figure 2*c*,*d*). In others, the cusp is enlarged and erect, having serrations in some taxa (figure 2*e*). Upper teeth of these strongly heterodont taxa are smaller than lowers, and are more similar to teeth of unrelated sharks such as the Scyliorhinidae, than *Squalus*, with a bilobed root and a crown with multiple (figure 2*c*) or single (figure 2*d*,*e*) cusps. Unlike the lower teeth of the same taxa, or any teeth of *Squalus*, the upper teeth clearly demonstrate an alternate arrangement (figure 2*c*–*e*). In some genera (*Aculeola*, *Centrosyllium* and adults of *Miroscyllium*), teeth very similar to those present in the upper dentitions of the related genus *Etmopterus* are present in both jaws, with all teeth showing a clear alternate pattern. The extreme form of this ‘secondary homodonty’ is present in *Trigonognathus* (figure 2*f*), where teeth are widely spaced and awl-like and strongly divergent from the pattern of *Squalus*.

The jaw cartilages of most Squaliformes are linguo-labially flattened, in contrast to most other sharks and rays where cartilages are rounded or oval in section (e.g. figure 4*a*). In the majority of sharks, teeth from several successive rows are in functional position at the same time, especially in taxa with low-crowned or small teeth. The flattened jaw cartilages in taxa such as *Squalus* cause replacement teeth to lie flat against the lingual face of the cartilage, rotating rapidly into functional position upon reaching the cartilage margin (figure 4*b*). As a result, only one or two rows of teeth are present along the jaw margin at any time. Increased compression and expansion of the root of lower teeth of more derived squaliforms is associated with increased compression of the lower jaw (Meckel's) cartilage (figure 4*c*). As a result, only a single functional row of teeth can be accommodated on the jaw margin at any time. By contrast, upper teeth are typically arranged on a curved jaw surface, allowing a number of teeth in one file to be functional (figure 2*e*). A number of rows of developing teeth are present on the lingual face of the jaw cartilages prior to their movement into a functional position. This is especially evident in the lower jaw of the most strongly heterodont species where the deep lingual face of the cartilage is covered in teeth of varying developmental states (figure 4*d*).

As the teeth of many squaliforms lock together to form a continuous cutting edge, the functional integrity of the dentition is likely to be compromised if teeth were to be shed randomly from the jaw margin. Teeth are shed as whole or partial tooth rows rather than as individual teeth, as the entire functional tooth row moves as a unit away from the jaw margin. The tooth row descends labially from the lingual face of the jaw before being lost, allowing a replacement set of teeth to move into position.

Within all of the taxa studied, teeth were among the first mineralized structures to develop within the embryo (figures 4*e* and 5*f*,*i*). Denticles and parts of the skeleton then mineralize simultaneously, with skeletal mineralization initiating in the jaw cartilage, hyoid and, in some species, the vertebral centra. By birth, the jaw cartilages and associated branchial cartilages are strongly mineralized. In *Etmopterus spinax*, the chondrocranium is only partially mineralized at birth (figure 4*f*), with the anterior portion remaining unmineralized into adulthood (figure 4*g*). Therefore, the teeth of at least some squaliforms function without the requirement for a rigid chondrocranium. The jaw suspension of squaliforms is orbitostylic, connected to the chondrocranium is the hyoid arch and more anteriorly placed ligaments, allowing significant movement of the jaws relative to the chondrocranium ([16] and references therein). Jaws, therefore, have to function without bracing against a mineralized skull and the majority of bite force has to be generated within the jaws. The unique cutting dentition seen within many squaliforms may therefore be a mechanism to generate an effective cutting bite without strongly braced jaws. While *Squalus* is a generalized feeder using both ram feeding and suction [16], many other squaliforms have a dentition most suitable for cutting sections of large prey items, culminating in the ectoparasitic feeding ability of *Isistius* [17].

## 6. Tooth development in *Squalus*

Four embryos of three species of *Squalus* were studied: two specimens of *S. acanthias*, one of *S. blainville* and a third referred to here as *Squalus* sp. (figure 5*a*–*h*).

### 6.1. *Squalus acanthias* and *Squalus blainville*

Tooth development in these two species was essentially identical. Of the two specimens of *S. acanthias*, the larger (14 cm) specimen showed a lower degree of development (no mineralized denticles or cartilage, fewer tooth rows) than the smaller (11 cm). The stage of dental development in *S. blainville* is very similar to that of the 11 cm *S. acanthias* specimen (figure 5*d*), but the dentition being more clearly demonstrated in the *S. blainville* specimen due to some degree of crushing of the *S. acanthias*. The embryo of *S. blainville* (figure 5*a*–*c*) shows a number of replacement teeth in files beneath the oral mucosa on the lingual face of the jaw cartilages (figure 5*a*,*b*) repeated along the jaw. The first-formed teeth have reached the jaw margins and more distally, have moved onto the occlusal edge. In addition to multiple rows of developing teeth, the specimen shows partial mineralization of the jaw cartilages and an incomplete covering of mineralized dermal denticles. The X-ray dense eye lenses are also conspicuous. The 14 cm *S. acanthias* specimen (figure 5*e*,*f*) also has numerous files of teeth, but the oldest (first teeth to form) have not yet moved to the jaw margins. There are no other mineralized structures in the head of this specimen.

**Figure 6.**
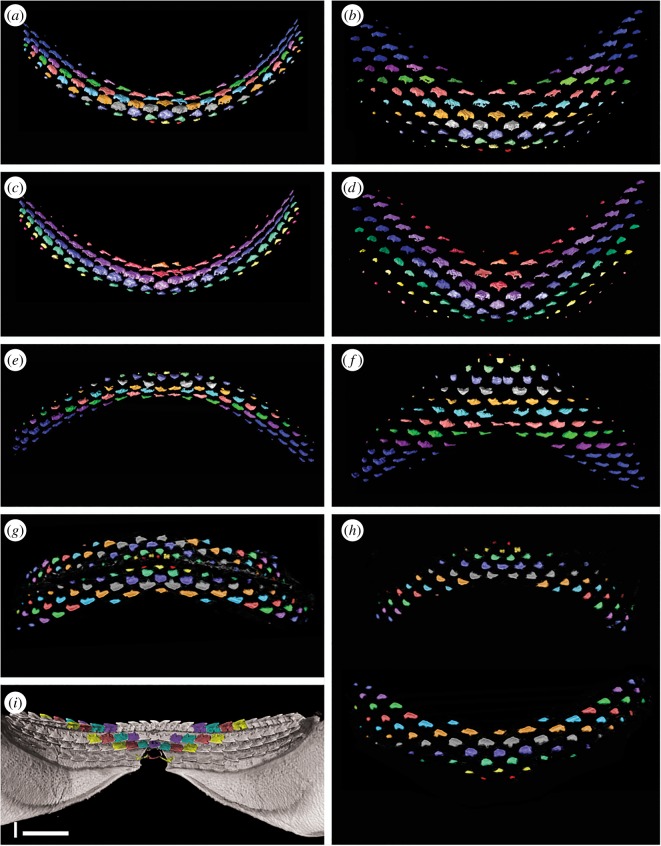
Segmented dentitions of *Squalus.* (*a*–*f*) Renders of the embryo of *Squalus blainville*. (*a*) Lower dentition colour coded to show alternate dentition pattern. (*b*) Exploded view of (*a*) to show pattern with greater clarity and remove the artefacts of jaw curvature. (*c*) Lower dentition colour coded to show oblique (functional) rows. (*d*) Exploded view of (*c*) to show pattern with greater clarity and remove the artefacts of jaw curvature. (*e*) Upper dentition colour coded to show alternate dentition pattern. Note lack of symphyseal teeth in all but row 2. (*f*) Exploded view of (*e*) to show pattern with greater clarity and remove the artefacts of jaw curvature. (*g*,*h*) Renders of the 14 cm embryo of *Squalus acanthias*. (*g*) Upper and lower dentition colour coded to show alternate dentition pattern. (*h*) Exploded view of (*g*) to show pattern with greater clarity and remove the artefacts of jaw curvature. (*i*) Lingual view of lower jaw of adult *Squalus acanthias*, colour coded to show the primary alternate dental pattern; oblique (functional) rows are now parallel to the jaw margin. Scale bar 10 mm.

While the total number and positions of teeth are distinct in the CT-scan images, curvature of the jaws and migration of teeth over the jaw margin make the relative developmental positions of teeth difficult to assess. Therefore, teeth were segmented out of the raw data so that tooth rows and files could be studied more clearly (figure 6). This allowed teeth to be spread as if they were on a flat surface rather than the curving inner surfaces of the jaw cartilages.

In unmodified images of the developing teeth (figure 5*d*,*f*), the pattern of tooth succession differs from that in the adult, and instead an alternating pattern of replacement teeth occurs, particularly closer to the symphysis (distally). The tooth series within these SAT units indicates that their numbers increase, as well as along the jaw by addition of new proximal files to each successive row. In upper and lower jaws of both specimens, the first teeth to mineralize are the parasymphyseal, on either side of the jaw symphysis (figure 6*a*,*b*, *e*–*h*, red). We interpret these as also representing the first teeth to form in the dentition, with an initiator symphyseal tooth being absent. The first symphysial teeth to mineralize are lingual to the first parasymphysial teeth, in a row comprising the symphyseal tooth and a tooth on either left and right side (figure 6*e*–*h*, yellow) or, in the lower jaw of the larger specimen, a row of six teeth distributed asymmetrically across the midline (figure 6*a*,*b*, yellow). As in alternate tooth replacement patterns observed elsewhere (e.g. [10,11]), symphyseal teeth are then present in every other tooth row. An exception to this is seen in the lower jaw of the 14 cm specimen (figure 6*e*,*f*), where a symphyseal tooth is present in the second tooth row, but absent, and its expected position left as an unfilled gap, in subsequent tooth rows. This appears to be an example of variable development in this specimen and not typical of the species, although the absence of a symphyseal tooth in a jaw of an adult *S. acanthias* (CJU reference collection specimen) suggests this condition may not be especially rare.

Teeth are added sequentially within each SAT unit, but also with one tooth added per row, setting up new tooth files proximally (figure 6*b*,*f*,*h*). Comparison with the tooth file count in adults suggests that the *S. blainville* embryo had the complete number disto-proximally, such that by birth there was no further development of proximal tooth files. In *S. blainville*, eight rows of well-mineralized teeth are present associated with the symphyseal regions of both lower and upper jaws. However, in more proximal positions, teeth are present representing successive tooth files (in developmental rows) with teeth of up to row 15 being present (figure 6*a*,*b*,*e*,*f*). In the 14 cm *S. acanthias* specimen, about six tooth rows are represented near the symphysis, but teeth representing up to 11 rows are present in more proximal positions (figure 6*g*,*h*). The initiation and development of files along tooth rows is therefore not synchronous with growth, with teeth representing parts of the later successive tooth rows developing initially in the proximal part of the jaw. This asynchroneity of tooth development across tooth rows has not been observed in other elasmobranchs (e.g. [10,11]). As discussed further below, this results in teeth achieving single functional rows at the jaw margins, but comprising teeth from different developmental rows (same colour, oblique, rows; figure 6*c*,*d*).

### 6.2. *Squalus* sp.

This specimen, from Uruguay, was mislabelled as *S. acanthias*, and is of an earlier developmental stage than the smaller *S. acanthias* and the *S. blainville* mentioned above, with little mineralization of jaw cartilages and no skin denticles (figure 5*g*,*h*). All teeth are more widely spaced than in the other specimens, with the oldest still present on the lingual jaw surface (figure 5*h*). The jaws show a strong degree of curvature, which inhibits the interpretation of the tooth development. In addition, the upper dentition partly overlies the lower, with the earliest teeth from the symphyseal regions of both jaws difficult to separate. It appears that, in the lower jaw at least, the first tooth row comprises five teeth, including the symphyseal tooth (figure 5*h*, red). After this, subsequent alternate rows develop as seen in *S. acanthias*, with progressive addition of additional teeth (new proximal tooth positions) away from the symphysis. As in the other specimens of *Squalus*, teeth in the proximal files develop asynchronously before those in the same developmental row closer to the symphysis, resulting in the formation of what will become functional oblique occlusal rows including teeth of sequential developmental rows.

## 7. Interpretation of tooth development in *Squalus*

The dentition of the early ontogenetic stages of *Squalus* is reminiscent of that seen in the shark *Scyliorhinus* [18] and batoids [11] but with some very significant differences that cannot be satisfactorily explained by previous tooth addition models. The first developmental tooth row in *S. acanthias* and *S. blainville* embryonic specimens comprises only two parasymphyseal teeth, with no mineralized symphyseal tooth being present (i.e. no symphyseal initiator tooth is present). This is a pattern previously recognized in batoids [11] but not sharks [10,18]. The specimen of *Squalus* sp. appears to differ from that of the other *Squalus* in possessing an odd number of teeth, including a symphyseal tooth, in the first developmental row (figure 5*h*, red). This variation in tooth number in the first developmental row, and in whether a symphyseal tooth is present, has previously been recognized in skates [11]. Subsequent alternate developmental tooth rows add teeth at a rate that appears to be very regular, with one additional tooth position per (developmental) row. This continues until perinatal stages, by which time the adult tooth count has been achieved. Within the Batoidea, this pattern of addition until a fixed ontogenetic stage is seen in *Myliobatis* but not *Discopyge* [11]. While the stepwise addition of tooth files with successive rows seen in embryonic *Squalus* is also observed in some batoids, the asynchroneity of proximal to distal development of teeth within a tooth row has not been observed within other elasmobranchs.

Within the alternate dental pattern of most elasmobranchs, including the embryos of *Squalus*, teeth are aligned in oblique tooth rows (figure 3) in addition to the alignment along developmental tooth rows. Oblique tooth rows connect teeth of various positions relative to the jaw margin, and as a result include teeth that are not simultaneously in a functional position and thus have no functional significance. Within the early ontogeny of *Squalus*, however, tooth position and alignment undergoes significant changes, eventually resulting in the adult *Squalus* arrangement. Comparison of tooth alignment in *Squalus* specimens of different ontogenetic stages allows this process to be understood, with oblique tooth rows of embryos being homologous with the tooth rows parallel to the jaw margin in the adult. The change in orientation of the tooth rows during *Squalus* ontogeny is facilitated by the asynchronous development of teeth along the jaw, as teeth produced at the same time will represent ‘later’ tooth rows in more proximal jaw positions. Tooth initiation along an oblique tooth row is therefore close to synchronous. Thus, while development of the first teeth within the jaw follows the pattern seen in alternate elasmobranch dentitions elsewhere, with teeth of the same row forming simultaneously, this rapidly changes during ontogeny. More rapid production of teeth in more proximal parts of the jaws facilitates the gradual rotation of oblique tooth rows towards parallelism with the jaw margin. Teeth therefore become aligned not along the developmental row as in other chondrichthyans, but along an oblique row, in which adjacent teeth actually belong to subsequent developmental rows, and not from the same developmental row as is seen in other elasmobranchs.

These obliquely aligned teeth are, however, not parallel to the jaw margin in embryos (figure 5*d*,*f*) but are in adults (figures 2*a* and 6*i*), forming functional rows that move onto the jaw margin as a single unit. In this process, a number of early formed distal teeth have to be shed prior to birth to generate a complete first functional tooth row. While the formation of teeth and their subsequent loss without use is wasteful of resources, it is necessary to generate the ‘saw blade’ dentition in *Squalus* by modification of the alternate developmental pattern. Adjacent teeth on the jaw margin of an adult *Squalus* are therefore within a functional row comprising teeth from many oblique developmental rows, and are not formed from a synchronous developmental row. The slight overlap of teeth within the alternate developmental rows is preserved as overlap in teeth within the functional row, ‘locking’ the teeth together. This overlapping pattern has been noted in other squaliforms [18] and termed ‘imbricate overlap’ [19].

## 8. Tooth development in other squaliforms

A single embryo of the partially heterodont centrophorid *D. calcea* was studied, along with embryos of the highly heterodont genera *Centroscymnus* and *Etmopterus*.

### 8.1. Deania calcea

The Centrophoridae possess a dentition morphologically transitional between *Squalus* and the highly heterodont squaliforms (see above; figure 5*i*,*j*). Relatively few teeth are mineralized within the upper dentition. Despite this, the alternate pattern seen in *Squalus* embryos is evident, at least near the jaw symphysis (figure 5*j*); this is also revealed by dissection. Teeth of more proximal parts of the jaw are incompletely resolved and it is not clear from scan renders or dissection of an additional specimen whether the first tooth row contains a symphyseal tooth. There is, however, evidence of file increase within successive tooth rows, as seen in *Squalus*. The dentition of the lower jaw is more clearly resolved, with a higher degree of mineralization of the teeth. This higher degree of mineralization was confirmed by dissection of a second embryo from the same brood. The upper dentition is asymmetrical, with teeth of different rows developed to different degrees on either side of the jaw. In the lower dentition, however, this is not the case. Teeth are aligned in clear parallel rows, each with a symphyseal tooth. The tooth rows are clearly equivalent to the pattern in upper dentitions of *Squalus* and *Deania* adults, but not *Squalus* embryos, nor the (symphyseal) *Deania* embryo upper dentition.

### 8.2. Centroscymnus coelolepis

**Figure 7.**
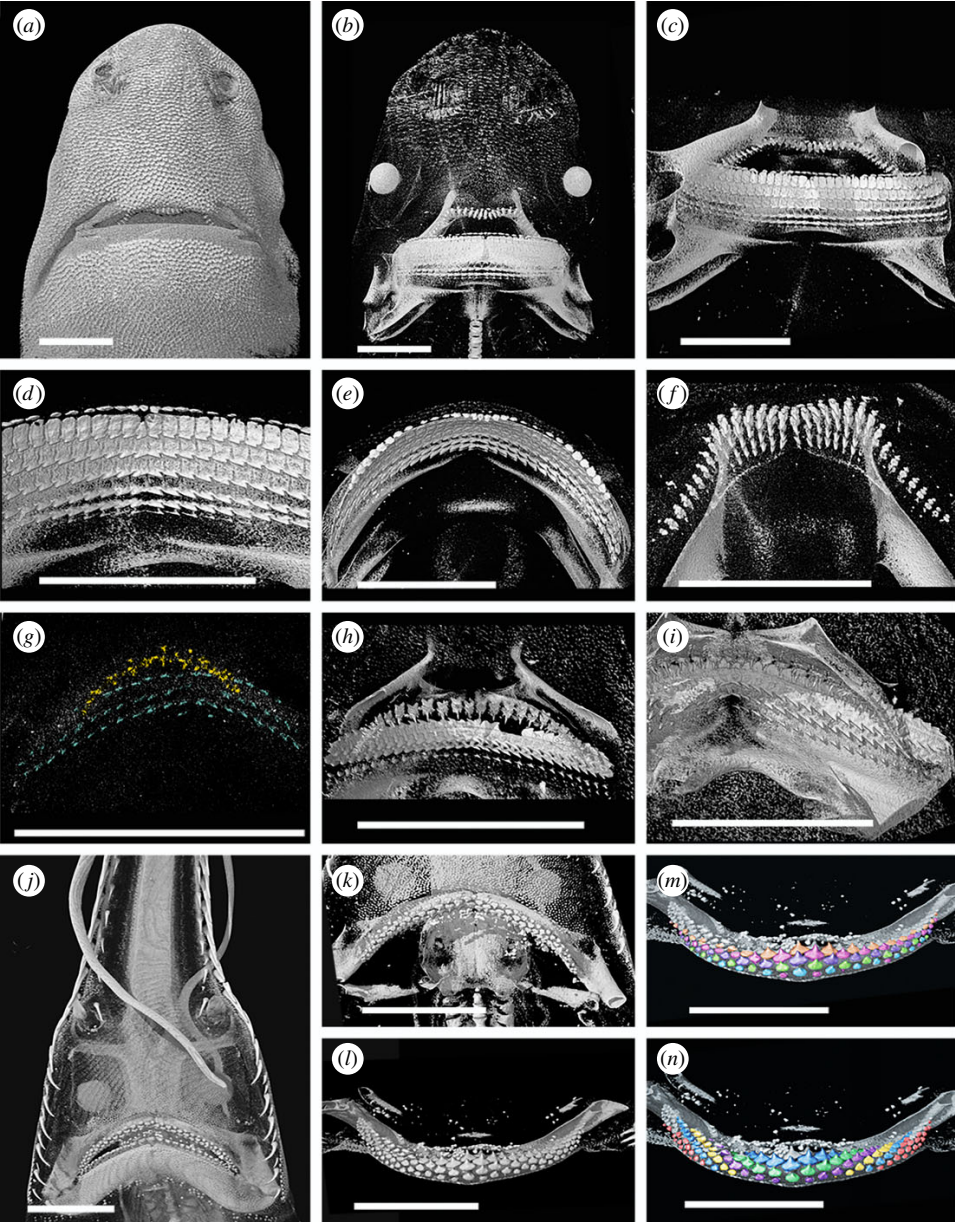
Tooth formation in heterodont squaliforms and *Pristiophorus*. All images rendered micro-CT data. Scale bars all 10 mm. (*a*–*f*) Renders of a 26 cm embryo of *Centroscymnus coelolepis*. (*a*) General view of ventral side of head. (*b*) General view of ventral side of head with greater penetration. (*c*) Lingual view of dentition. (*d*) Detail of lingual view of lower dentition. (*e*) Occlusal view of lower dentition showing the first-formed tooth row. (*f*) Occlusal view of upper dentition. (*g*) Lingual view of the dentition of a 10 cm embryo of *Etmopterus spinax*; the mineralized tips of the upper teeth (yellow) and lower teeth (blue) can be seen. (*h*) Lingual view of the dentition of a 16 cm juvenile of *Etmopterus spinax*. (*i*) Lingual view of the dentition of an adult of *Etmopterus spinax*. (*j*–*n*) Renders of an embryo of *Pristiophorus cirratus*; Australia. (*j*) General view of ventral side of head. (*k*) Occlusal view of upper dentition. (*l*) Occlusal view of lower dentition. (*m*) Lower dentition colour coded to show alternate dental pattern. (*n*) Lower dentition colour coded to show oblique rows, in this case not forming functional ‘blades’ as in squaliforms.

In the embryo of *Centroscymnus coelolepis*, CT-scan renders revealed several rows of lower and upper teeth. Some upper teeth were visible on the outer jaw surface (figure 7*a*,*b*), but no lower teeth could be observed in their functional position. The larger lower teeth were more clearly rendered and their relative positions more readily assessed. The lower tooth rows are parallel to each other and the jaw margin, and all rows possess a symphyseal tooth. Seven rows of teeth are visible within the lower jaw, with the oldest three being fully mineralized along the length of the jaw (figure 7*c*,*d*). All but the first tooth row are composed of teeth very similar in shape to those in the adult. The first tooth row (figure 7*e*) comprises small, ovoid teeth with no differentiated cusp. These are present on the jaw margin but have not yet reached functional position, being partially rotated at the jaw margin. As teeth in *Centroscymnus* are not shed until replacement teeth are in functional position, it is evident that no older tooth row had been present and subsequently lost. The teeth in the first row are of uneven size and distribution; they are larger and more closely spaced near the symphysis and more proximally along the jaw, with smaller, more widely spaced teeth between these, giving the impression of a diastema. This is in contrast to the similar size and distribution of subsequent teeth. There are also fewer teeth in the first tooth row than in subsequent rows. Teeth in the first row are approximately aligned with those in subsequent rows, and there is no evidence that they alternate with teeth in the second row. As in *Deania*, this is more comparable to the adult *Squalus* condition.

The upper dentition is very similar to the adult (figure 2*d*), with large numbers of slender and close-packed teeth (figure 7*f*). These show an alternate pattern but the degree of resolution and strong curvature of the jaws makes this difficult to assess. While minute teeth of the first rows are clearly present, the relative sizes of adjacent first teeth cannot be reliably addressed from the scan data present.

### 8.3. Etmopterus spinax

The only embryo of *E. spinax* studied represented a relatively early ontogenetic stage with poorly developed teeth that were difficult to render (figure 4*e*). Despite this, the enameloid tips of teeth could be resolved and provide informative clues to tooth development (figure 7*g*). In the lower jaw, three tooth rows can clearly be seen, all at a similar stage of mineralization. As with the lower dentition of the embryo of *C. coelolepis*, the tooth rows do not alternate and a symphyseal tooth is present in each row. As several tooth rows show the same degree of (incomplete) mineralization, several teeth rows must have initiated synchronously within the dental lamina. The number of replacement tooth rows present within the lower jaw appears constant throughout ontogeny, with three rows in the embryo, juvenile (figure 7*g*) and adult (figure 7*i*). The upper teeth are far less well resolved. Upper teeth of *E. spinax* are multi-cusped, in contrast to many other squaliforms. Some of the poorly mineralized upper teeth appear to show the presence of multiple mineralized cusps, while mineralization of the remainder of the tooth is lacking. As with the lower dentition, there appear to be several tooth rows present, especially close to the jaw symphysis. The relative position of mineralized tooth cusps suggests the upper teeth are arranged in an alternate pattern.

## 9. Interpretation of tooth development in heterodont squaliforms

Dentitions of the more heterodont squaliform taxa appear varied and different from the dentition in *Squalus*. Upper teeth of both *Centroscymnus* and *Etmopterus* embryos form an alternate pattern as is seen in adults of the same taxa, and most other elasmobranchs. By contrast, lower teeth of *Deania*, *Centroscymnus* and *Etmopterus* are arranged in rows parallel to the jaw margin that do not alternate; all possess a symphyseal tooth and form a single file arrangement (*sensu* [10]). The upper dentition in *Deania* embryos most closely resembles embryos of *Squalus*, with the dominant alignment of teeth suggesting a single file pattern, but with an alternate pattern also being visible, especially near the symphysis. In upper dentitions of *Deania*, as in *Squalus*, single file tooth replacement is a developmental modification of the inherited alternate pattern characteristic of other elasmobranchs.

The embryonic lower dentitions of *Deania*, *Centroscymnus* and *Etmopterus* do not show an alternate pattern but rather a single file pattern as seen in adults of *Squalus* and upper dentitions of adult *Deania*. As these lower jaw dentitions are essentially identical, it is unlikely that a different developmental mechanism generates these single file dentitions. It is therefore probable that tooth rows within lower dentitions of these heterodont taxa also represent modified oblique tooth rows. The absence of clearly alternate teeth within initial stages of development of the embryonic dentition is most probably explained by an accelerated development of the ‘adult’ tooth arrangement in the first teeth to mineralize, already aligned into jaw margin parallel oblique rows. As a result, teeth are arranged in functional, rather than developmental, rows from their initiation.

## 10. Tooth development in the Pristiophoridae

The Pristiophoridae represent an outgroup within the Squalea to the squaliforms. Adults have low-crowned teeth showing an alternate pattern of tooth replacement (e.g. [13], figure 6*a*). Jaw cartilages are wide and several rows of teeth are in a functional position at any point in time. Embryos of *Pristiophorus cirratus* and *Pliotrema warreni* were studied. The latter was of an earlier growth stage with only a small number of teeth present, revealing no additional information to that more clearly observed in *Pristiophorus*.

The dentition in the *P. cirratus* embryo resembles that of the adult, with an obvious alternating dentition on both upper and lower jaws (figure 7*j*–*l*). The initial tooth row within both jaws comprises a pair of parasymphyseal teeth (figure 7*m*, red), although an additional tooth in a more lateral position also appears to be present on one side of the lower dentition. The second row of teeth within the lower dentition includes a symphyseal tooth and six files of proximal teeth. Subsequent tooth rows contain additional teeth, with one additional proximal tooth file being added on each side of the jaw with each successive row. As a result of developmental timing and alternate patterning, teeth within the embryonic dentition of *Pristiophorus* become aligned in oblique sets, each comprising developmentally younger teeth formed at successive times (figure 7*n*). The pattern order of embryonic dentitions in *Pristiophorus* and *Squalus* are identical, but that of adults is not, with *Pristiophorus* lacking the asynchronous tooth development present in *Squalus*.

## 11. Pristiophorid dentitions as a key to dental homology in squaliforms

**Figure 8.**
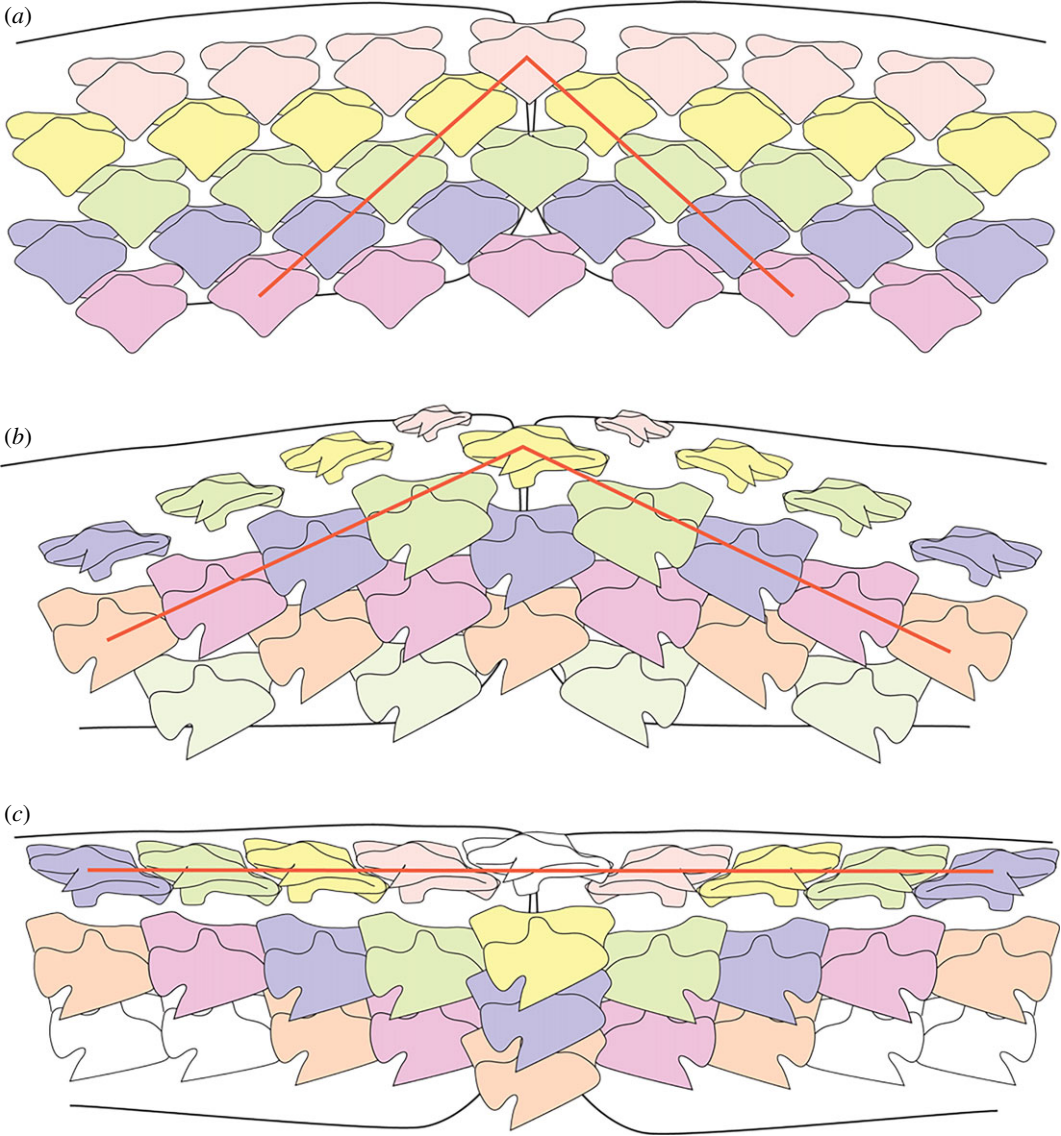
Diagrammatic representation of tooth patterning showing the position of oblique tooth rows. (*a*) *Pristiophorus*, with an alternate dental pattern. (*b*) Embryonic *Squalus* with an alternate dental pattern and progressive addition of tooth files with new tooth rows. Oblique tooth rows are not parallel to the jaw margin. (*c*) Adult *Squalus* showing oblique tooth rows parallel to the jaw margin.

The pristiophorids and the squaliforms are sister taxa (figure 1), but have teeth that are morphologically and functionally very different. As a result, similarities in the development of the dentitions of pristiophorids and the squaliforms are due to common ancestry and not convergence due to functional constraints. The upper dentition of embryonic and adult *Centroscymnus* and *Etmopterus* shows an alternate pattern, as in the pristiophorids, despite extreme differences in tooth morphology, indicative of a similar developmental mechanism. Similarity between the dental arrangement in *Pristiophorus* and embryonic *Squalus* again indicates that alternate tooth replacement and addition is common to both. The adult dentition of *Squalus*, with obvious single file tooth replacement, differs from the pattern seen in *Pristiophorus* and embryonic *Squalus*, but is generated from the alternate pattern during ontogeny. While the tooth replacement pattern in adult *Squalus* and the lower dentitions of embryonic and adult *Deania*, *Centroscymnus* and *Etmopterus* is single file and differs from the *Pristiophorus* dentition, all develop from the alternate pattern formula (figure 8).

Comparisons of the dental development of *Pristiophorus* and *Squalus* demonstrate how the single file tooth replacement pattern in *Squalus* can be generated from an alternate file replacement pattern by the formation of oblique rows which end as a functional row with sequential timing along the jaw. It is only in embryos of *Squalus*, and upper dentitions of *Deania*, that an intermediate dental geometry between the alternate pattern of *Pristiophorus* and the single file pattern of most squaliforms is seen and allows the homology of dental development to be observed.

## 12. Discussion

The adult dentitions of many squaliform sharks differ from those of most other elasmobranchs in possessing a well-developed single file tooth replacement pattern. Despite this, an alternate pattern is present in the upper dentitions of many species, and the lower dentitions of a small number of homodont species. Squaliforms therefore appear to possess two very different tooth replacement patterns within the clade, and in many taxa, within an individual. It is evident that a great deal of plasticity of tooth development is present within the group. Understanding the nature of this plasticity, and homology within squaliform dentitions, is critical to evaluation of the plesiomorphic state of dental development within the squaliform sharks and the elasmobranchs more generally.

A universal developmental model has been suggested [8] for all jawed vertebrates but here we have focused on one major clade of gnathostomes, the Chondrichthyes. Chondrichthyans are the only cohort that does not have any dermal bone within the cartilaginous jaws, nor over the whole body, so that there is not a bone of dental attachment. They also exemplify the classic dental lamina [20] as first proposed by Reif [12]. As such this is the only oral developmental system where the production of all teeth, including first initiated and successor teeth, is confined to this epithelial folding that tucks in lingually [10] within the cartilage furrow of both jaws. Epithelial connectivity within the dental lamina facilitates relocation of the odontogenic stem cells (Sox2+) that have the potential to migrate within the dental epithelium. Genetic fate mapping demonstrates their presence in the mammalian primary dental lamina and successional dental lamina [21], as well, that serially added molars in the mouse form from Sox2+ cells [22]. This is the only place in developing mammalian dentitions where a comparable dental lamina is present, but these are serially iterative teeth along the proximal jaw, rather than replacement teeth (none are replaced in the mouse dentition). This could be a common mechanism and chondrichthyans are an excellent model to test this, where initiator teeth are formed serially along the jaw from distal to proximal, but followed by successor teeth in each file, as these form from a continuous dental lamina.

It was suggested [8] that all toothed fields in the chondrichthyan dentition start from a pioneer tooth at the jaw symphysis and that this autonomously regulates the patterning of tooth addition along the row (distal to proximal), and all successive replacement teeth. Two types of tooth patterning were identified, both generated from this initiator tooth and we have shown how that pattern unfolds differently. The iterative sequence addition model, based on a clonal generative unit comprising two adjacent tooth files, is based on alternate timing and arrangement, and proposed to explain the development of the alternate tooth pattern [8]. Alternatively, in the single file tooth pattern, each file would be one generative set (clonal derivatives) and iterative, producing distal to proximal tooth files along the jaw.

Examination of squalean embryonic dentitions has revealed that the single file tooth pattern is not generated from these types of identical tooth files, but from modification of an alternate pattern. In the embryonic dentition of *Squalus* and *Pristiophorus*, an alternate tooth development pattern can be identified, initiating from the symphyseal region. However, only a small number of the tooth file pairs described in the SAM are present near the symphysis, with a notable change in timing of tooth development producing more teeth proximally along the jaw. Further modification of this pattern in *Squalus*, with loss of earlier-formed teeth, has the effect of aligning teeth in oblique rows comprising teeth of successive developmental rows. Eventually, these oblique rows form single functional rows of teeth along the jaw margin, a pattern that persists in adults. Therefore, the single file tooth replacement pattern characteristic of the Squaliformes derives from a modified embryonic alternate pattern.

**Figure 9.**
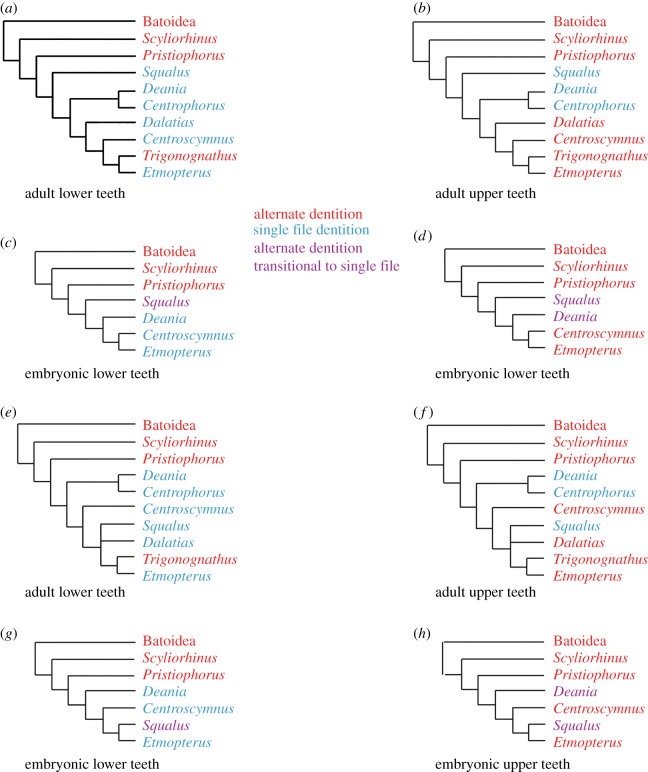
Tooth development patterns of adult and embryo squaliform taxa plotted against the phylogenetic models of figure 1. (*a*–*d*) The phylogeny of Straube *et al.* [5] showing dental arrangements of lower (*a*) and upper (*b*) teeth of adults and lower (*c*) and upper (*d*) teeth of embryos. (*e*–*h*) The phylogeny of Naylor *et al.* [3] showing dental arrangements of lower (*e*) and upper (*f*) teeth of adults and lower (*g*) and upper (*h*) teeth of embryos. Dental patterns colour coded as alternate (red), single file (blue) and transitional (purple) dentitions. Squalean taxa not included in the current study are not considered.

Alternate dentitions are present in all batoids and galean sharks studied to date [10,11] and it therefore seems likely that the development of (functional) single file dentitions is a derived state limited to the squalean sharks. Within this clade (figure 9), there are several families in addition to the Squaliformes that appear to possess single file tooth replacement. Teeth of *Chamydoselachus* and *Squatina* are widely spaced and tooth replacement cannot readily be assessed without study of embryos. In addition, the phylogenetic position of *Protospinax*, an extinct genus with alternating teeth, is uncertain [23]. Teeth of *Echinorhinus* are blade-like and superficially very similar to those of squaliforms; teeth show single file replacement similar to that of many squaliforms, but symphyseal teeth are absent. Embryonic dentitions of *Echinorhinus* are as yet unstudied. All three genera within the Hexanchidae have blade-like teeth along the majority of the lower jaw, although the situation of the smaller and more widely spaced upper teeth is more difficult to assess. In addition, much reduced teeth in the most proximal parts of the jaw within the Hexanchidae show a clear alternate pattern. It therefore appears that, if the alternate tooth replacement pattern is plesiomorphic to the elasmobranchs, a functional, if not developmental, single file pattern has evolved on up to three occasions within the Squalea.

The development of the *Squalus* dentition is intermediate between that of non-squaliform squaleans such as *Pristiophorus* and strongly heterodont squaliforms such as *Centroscymnus* and *Etmopterus*, with the heterodont taxa possessing direct development of the oblique functional rows, first seen in the embryonic stages (figure 9). *Deania* represents a position developmentally between *Squalus* and *Centroscymnus* + *Etmopterus*, with upper teeth developing more like those of *Squalus* and lower teeth more like those of *Centroscymnus* + *Etmopterus*. This developmental gradient of *Pristiophorus* to *Squalus* to *Deania* to *Centroscymnus* + *Etmopterus* is more parsimonious with respect to phylogenies recovering Squalidae as the most basal member of the Squaliformes and the strongly heterodont forms more derived [4–6], than others where Squalidae is highly derived [3]. Having the Squalidae as the basal family of the Squaliformes is also consistent with the fossil record [1,5,6] and tooth morphology [1].

The generation of functional rows of bladed teeth presenting at the jaw margin as a single, serrated blade is likely to have been one of the key innovations that allowed the squaliforms to become a very diverse and successful clade. The earliest (Early Cretaceous) fossil record of the squaliforms is restricted to the genus *Protosqualus*, which possessed teeth similar to those of *Squalus* [1,6]. This genus was at its most abundant in cool, and apparently nutrient-poor, waters (e.g. [24]), the same conditions commonly dominated by squaliforms today. The major radiation within the clade was probably only possible after the appearance of the strongly heterodont forms (e.g. [5,6]) in the Late Cretaceous. It is possible that the change in tooth development from that of *Squalus* to that of forms exemplified by *Deania*, *Centroscymnus* and *Etmopterus* contributed to this radiation. In *Squalus*, the initial alternate tooth arrangement has to be modified by the time of birth to allow the development of a cutting jaw margin. This requires a number of early formed teeth to be lost prior to birth to allow the oblique tooth sets to rotate into position, with tooth wastage losing energy, and potentially phosphate, from the developing embryo. This could have significant implications within low-nutrient environments. The development of teeth already aligned in functional rows in heterodont squaliforms removes this wastage. These squaliforms therefore had a highly efficient method of producing a very versatile dentition unlike that possessed by any other potential competition.

The unique tooth development pattern of most chondrichthyans allows both rapid and simultaneous replacement to maintain function of several teeth of the same file at the jaw margin. This dental lamina-controlled developmental pattern in chondrichthyans also mediates the development of extremely diverse dentitions, with tooth morphologies unknown within other gnathostomes. Within the squaliforms, there is considerable, and previously unrecognized, plasticity enabled by one shared chondrichthyan developmental mechanism. This combination of an intrinsic, flexible modifiable mechanism for tooth formation, and disposable teeth, has allowed the chondrichthyans to occupy a wide range of trophic niches that vastly outweighs the relatively small number of extant species within the clade.

## Data accessibility

Data available on the NHM Data Portal (http://data.nhm.ac.uk).
